# Design and usability of Abu Dhabi Food Intake24: a culturally adapted digital 24-hour dietary recall for the United Arab Emirates

**DOI:** 10.3389/fnut.2026.1866000

**Published:** 2026-08-20

**Authors:** Habiba I. Ali, Lamis Akkad, Minat Allah T. Al Hassan, Razan S. Almasri, Hassan Al Marzooqi, Hanan S. Afifi, Mohamed G. Shehata, Mo’ath F. Bataineh

**Affiliations:** 1 Department of Nutrition and Health, College of Medicine and Health Sciences, United Arab Emirates University, Al Ain, United Arab Emirates; 2 Food Research Section, Applied Research and Capacity Building Division, Abu Dhabi Agriculture and Food Safety Authority (ADAFSA), Abu Dhabi, United Arab Emirates

**Keywords:** digital dietary assessment, mobile applications, multiple pass method, online 24-h recall, system usability survey, United Arab Emirates

## Abstract

**Background:**

Standardized dietary assessment tools are essential for evaluating population diets, eating patterns, and their relationships with health. Although digital dietary assessment tools can support more standardized and efficient dietary data collection, they require appropriate cultural adaptation to be applicable in different populations. In the United Arab Emirates (UAE), the lack of a culturally appropriate digital 24-h dietary recall tool limits standardized dietary assessment for nutrition research and population surveys.

**Objective:**

To develop a culturally adapted digital 24-h dietary recall application for the UAE population’s eating practices.

**Methods:**

Abu Dhabi Food Intake24 (ADINTAKE24) is a tablet-optimized, Android-based application. It collects survey questionnaires and 24-h dietary recall data, calculates nutrient intake, and compares the results with the recommendations. The ADINTAKE24 uses the multiple-pass method that integrates food composition data from the United States Department of Agriculture (USDA), regional, and local sources. UAE-specific food photographs aid in estimating food portion sizes. The application is available in Arabic and English and includes a web-based administrative interface. The system was iteratively developed and tested under field conditions during a large community-based nutrition survey. A pilot survey with families informed system refinement, followed by usability assessment among 35 trained interviewers using the System Usability Scale (SUS).

**Results:**

After initial development, the tool was piloted with 98 participants from 39 households using an interviewer-administered approach. Challenges identified during the pilot study included problems with saving and searching for food items in the food composition databases on the server during field data collection. Refinements were made based on the tool’s feedback to improve its functionality. The mean System Usability Scale (SUS) score was 69.9 (95% CI: 66.0–73.9; SD = 11.6).

**Conclusion:**

Abu Dhabi Food Intake24 addresses a key gap in digital dietary assessment in the UAE. Further usability evaluation of the finalized application and validation for self-administered use are needed before wider implementation.

## 1 Introduction

Dietary assessment is an essential component in evaluating the nutritional status of individuals (1). Dietary data collection is a critical component of large-scale surveys aimed at studying diet-population consumption patterns at the regional or national level (2). These data serve as the foundation for policies and regulations aimed at improving the population’s nutritional status.

Common dietary assessment methods include 24-h recalls, dietary records, and food frequency questionnaires (3). Technological advancements and availability have led to the development of online dietary assessment tools for use worldwide (4), given their variable benefits. Documented benefits of online dietary assessment include reduced interviewer burden, easier data collection, and lowered processing costs, as well as standardizing the recall process, providing digital photographic portion estimation, and automatically matching foods to nutritional databases (5).

Online dietary assessment tools can be categorized into web-based tools, mobile applications (apps), and camera-based tools (6). For example, INTAKE24 is an online, web-based tool (7) that has been widely adopted in countries such as Scotland (8), Australia (9), and New Zealand (10), and has recently been adapted for South Asian countries (11). However, these tools have primarily been developed for European populations and European databases (7, 12) or for North American populations and databases (13).

Implementing these tools across different countries requires manually adding and matching culturally specific food items, which is less efficient and more burdensome, especially in large-scale surveys, unless significant effort is invested in culturally adapting them before use. Effective adaptation includes integrating population-specific data, not only on commonly consumed foods but also on portion sizes and recipes, to enhance relevance (14).

Recognizing the lack of culturally appropriate online tools in the Middle East, Bawajeeh et al. (15) developed an Arabic food composition database, primarily for Saudi Arabia and Kuwait. This database was incorporated into an online validation of the Arabic version of the dietary assessment tool, MyFood24, developed mainly for use in the United Kingdom population (12). However, it has since been translated into various languages, including German (16). Despite this progress, many nutrition and food consumption studies in the Middle East and the Gulf Cooperation Council countries, namely Saudi Arabia, Kuwait, the UAE, Qatar, Bahrain, and Oman, use different nutrition analysis software that primarily rely on the USDA database (17–19). Therefore, to benchmark the results of dietary collection tools, it may be more appropriate to use a tool that uses the USDA database to reduce variability (20).

In the UAE, El Mesmoudi et al. (21) developed and validated an online food-frequency questionnaire to assess dietary intake among Emirati adults. This tool was adapted from the Food4me online tool (22) and implemented as a self-administered, web-based Arabic version optimized for laptops. While food-frequency questionnaires have been preferred in large epidemiologic studies due to their feasibility, lower cost, and reduced administrative resources (23), 24-h recalls are generally more accurate at capturing dietary intake (24, 25).

Nowadays, mobile applications are increasingly used for dietary tracking, whether commercial or research-developed (26). These apps are mainly designed to meet the needs of their intended populations (27–29). A few commercial apps are usable in the UAE, such as MyFitnessPal, which includes international and Middle Eastern foods. It gives users the freedom to add custom food items and recipes to expand their database. However, the open-access nature of user-generated food entries may compromise database quality and reduce reliability for research applications. Moreover, evaluating the validity and reliability of digital dietary assessment applications is challenging because they differ considerably in the nutritional databases and food composition data they use, and information about these underlying data sources is often limited (26).

To date, most dietary data collection in the UAE has relied on traditional 24-h recall methods, requiring significant manual processing data (18, 30, 31). The lack of a culturally adapted digital 24-h recall tool limits the accurate capture of local eating patterns and practices. Currently, no 24-h digital dietary recall system tailored to the UAE population, integrating high-quality international and regional food composition databases, is available. This study aimed to develop Abu Dhabi Food Intake24, a culturally adapted digital 24-h dietary recall tool for assessing dietary intake, eating patterns, and food practices in the United Arab Emirates, and to evaluate its usability through pilot testing, system refinement, and user evaluation.

## 2 Methods

### 2.1 Design and development

Abu Dhabi Food Intake24 (ADINTAKE24) is a mobile-based 24-h dietary recall application developed to support the Abu Dhabi Food Consumption Survey, conducted by the United Arab Emirates University (UAEU) in collaboration with the Abu Dhabi Agriculture and Food Safety Authority (ADAFSA). The survey aims to collect detailed dietary and related information from members of a representative sample of 3,920 families living in the Emirate of Abu Dhabi, United Arab Emirates. To support standardized and efficient data collection, Abu Dhabi Food Intake24 was designed as the primary tool for capturing participants’ dietary intake over the 24 h preceding the interview.

The ADINTAKE24 platform consists of two main components: (1) a mobile application used by trained data collectors to administer the 24-h dietary recall and survey questionnaires, and (2) a secure web-based portal for centralized data storage, monitoring, and management. The system integrates survey questionnaires with the dietary recall component, enabling simultaneous collection of sociodemographic, anthropometric, and 24-h recall data. The application was developed through collaboration between researchers from the United Arab Emirates University and the Abu Dhabi Agriculture and Food Safety Authority in partnership with software engineers from a contracted technology company.

During the initial phase, system requirements and design specifications were established. The system was subsequently optimized for tablet devices due to their increasing use in dietary surveys, as they offer enhanced mobility, efficient data collection, and storage (32–34). Abu Dhabi Intake24 was designed to collect data in both English and Arabic.

#### 2.1.1 Technical implementation

The application was developed for Android. The Web Admin Portal was built using ReactJS. The Back-End Services were developed using ASP.NET 6. The storage databases used are SQLite for the app, which contains the preloaded food database, and MSSQL 20 for data storage in the web portal. The Abu Dhabi Food Intake 24 User Manual was created as a step-by-step guide to help users explore the application and its functionality.

The Abu Dhabi Food Intake24 incorporates preloaded food composition databases from multiple sources to ensure comprehensive and culturally relevant food coverage. The primary source is the United States Department of Agriculture FoodData Central database, 2024 (35) which provides nutrient composition data for an extensive range of foods. To improve regional relevance, the database was expanded to include 23 traditional Emirati composite dishes (36, 37), 75 Kuwaiti composite dishes (38–41), and 104 recipes collected from a representative sample of Emirati households. The system estimates dietary intake for 30 dietary components, including energy; macronutrients (carbohydrates, protein, total fat, saturated fat, monounsaturated fat, polyunsaturated fat, trans fatty acids, cholesterol, and total dietary fiber); vitamins (vitamin A [retinol activity equivalents, RAE], thiamin [vitamin B1], riboflavin [vitamin B2], niacin [vitamin B3], vitamin B6, vitamin B12, folate, vitamin C, vitamin D, vitamin E [α-tocopherol], and vitamin K); minerals (calcium, iron, magnesium, phosphorus, potassium, sodium, and zinc); caffeine; water from foods; and drinking water. In addition, participants report their drinking water intake and indicate whether their food intake on the recall day was usual or less than the usual amounts.

#### 2.1.2 24-h recall

**Figure 1:**
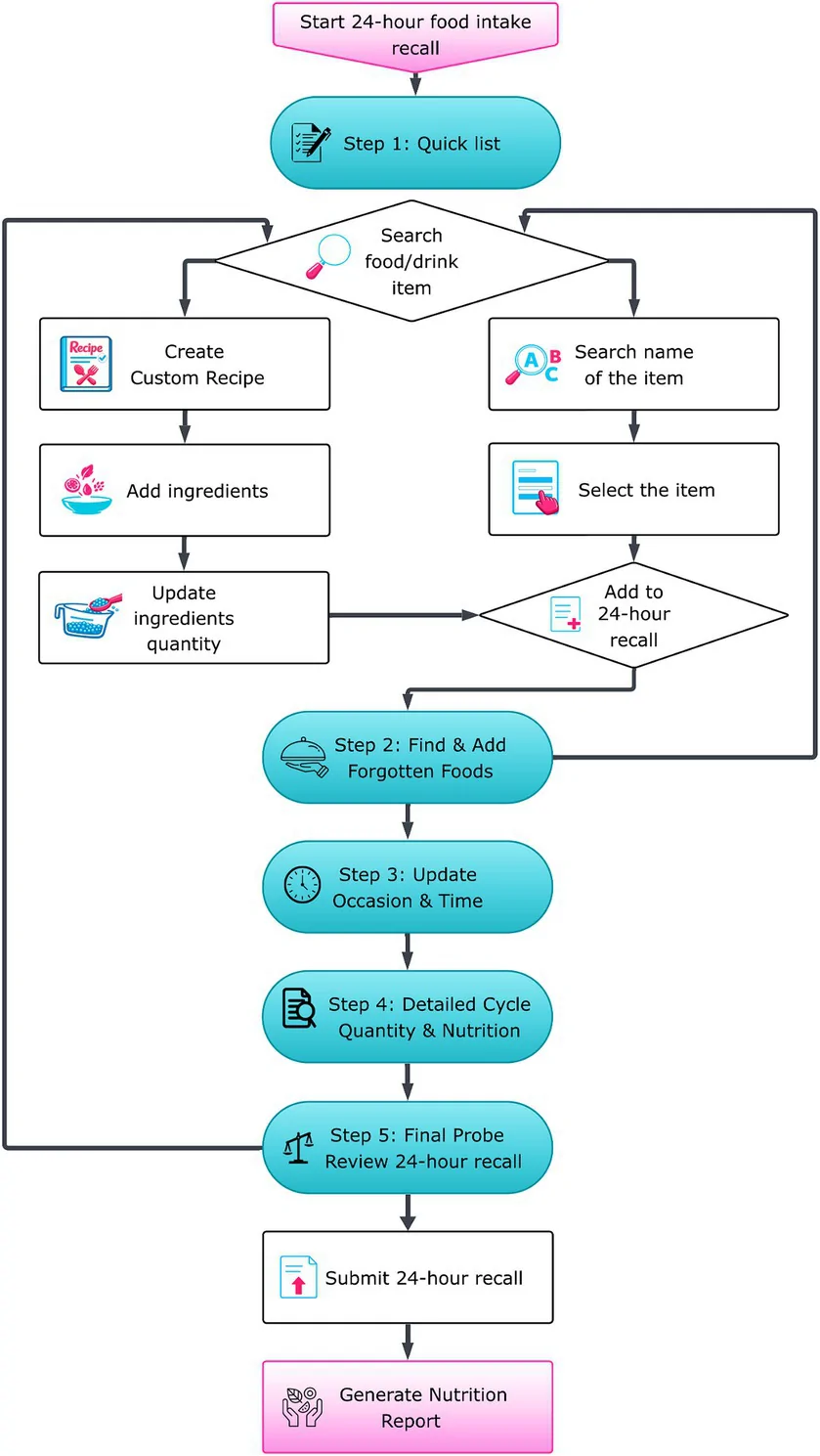
24 h dietary recall workflow in the application. The recall begins with the Quick List, where users search for and select foods from the food composition database or create a custom recipe by adding ingredients and their quantities. Selected foods and recipes are added to the 24 h recall. The application then guides users through the Find and Add Forgotten Foods step to prompt recall of commonly omitted items. Subsequently, users record the eating occasion and time for each food item, followed by the Detailed Cycle, during which portion sizes are estimated using standardized household measures, serving sizes, gram weights, and a validated UAE food photograph atlas. Finally, users review the completed recall in the Final Review screen, where additional foods can be added if needed, before submitting the recall. Upon submission, the application generates a nutrient intake report summarizing the estimated dietary intake.

The dietary recall was designed using the Multiple Pass method (42), as it is commonly used in online 24-h recall tools. It provides several advantages, such as improved dietary recall accuracy and reduced bias (43). Figure 1 summarizes the 24-h recall process steps adapted in the application, with the blue boxes showing the 5 steps of the multiple-pass method.

First, the user enters a quick list of all foods and beverages consumed in the previous 24 h, as shown in Figure 1, which is the first step of the multiple-pass method. Then the app prompts the user to recall whether they have consumed any common, forgotten foods, such as coffee, dates, or snacks. Consistent with the third step of the multiple-pass method, the user is prompted to provide contextual information for each food item, including time of consumption, eating location, and any activity accompanying it, such as watching television or using a mobile phone.

**Figure 2:**
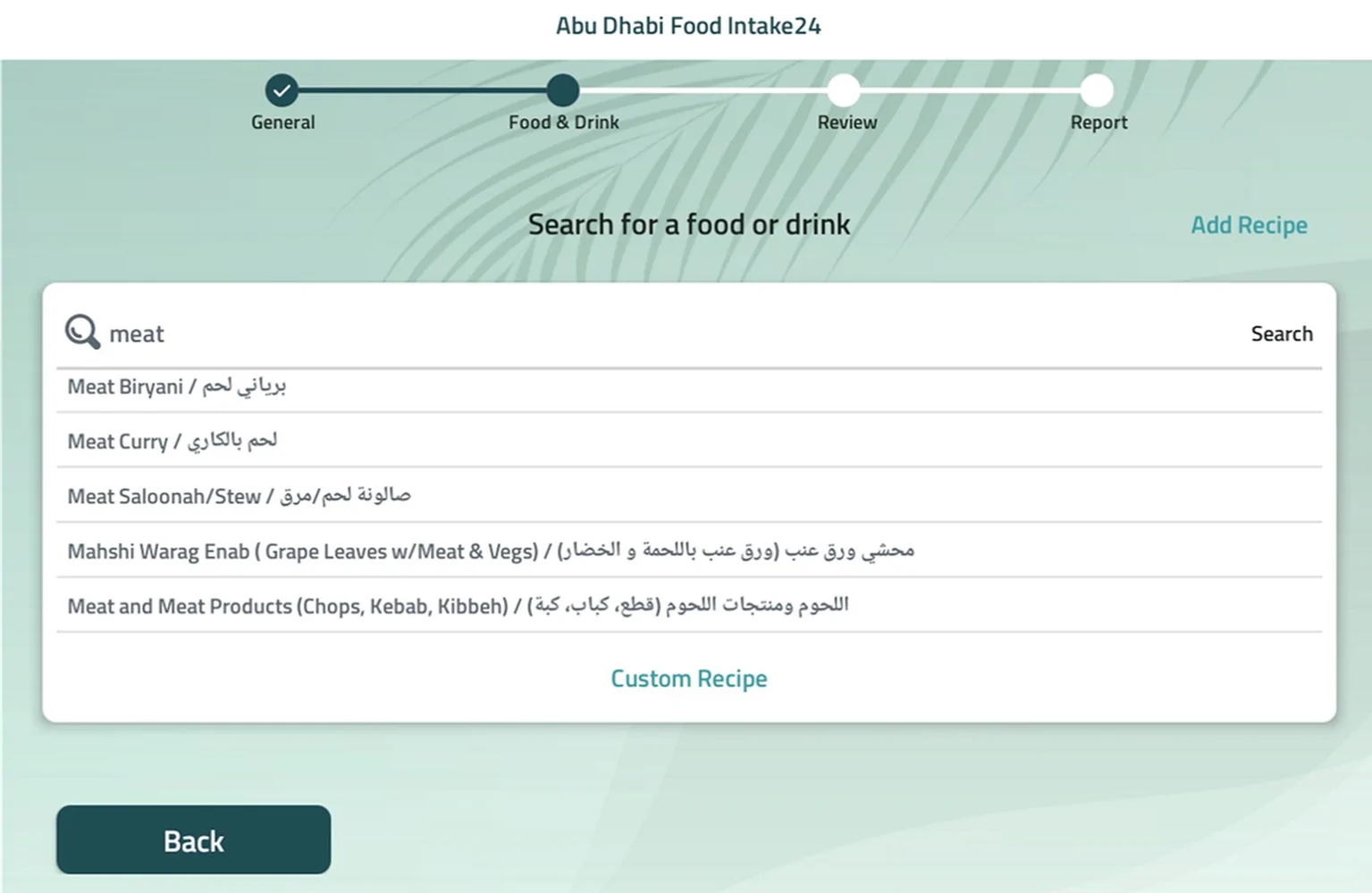
Food search interface in Abu Dhabi Food Intake24. The application allows users to search for foods and beverages by entering keywords into the search box. A list of matching food items from the integrated food composition database is displayed, from which the appropriate item can be selected and added to the 24 h dietary recall. Users may also select Custom Recipe to enter a composite dish by searching for and adding individual ingredients and their quantities. Figure has been reproduced from Abu Dhabi Food Intake 24, licensed under the Apache License, Version 2.0 (the “License”); you may not use this file except in compliance with the License.

The fourth step involves collecting detailed information on each reported food item, including preparation method and portion-size estimation, supported by the UAE Photographic Atlas of Food Portions, integrated into the Abu Dhabi Food Intake24 platform. The final step of the multiple-pass method provides a structured probe that prompts the user to add any additional items and presents a list of all reported foods for final review. The application allows the user to edit or delete entries before submission, thereby enhancing completeness and accuracy.

Users of the application can add foods and beverages to the 24-h recall by searching the integrated food composition database using keywords (Figure 2). The application displays matching food items from the integrated database, allowing users to select the appropriate item and add it to the recall.

**Figure 3:**
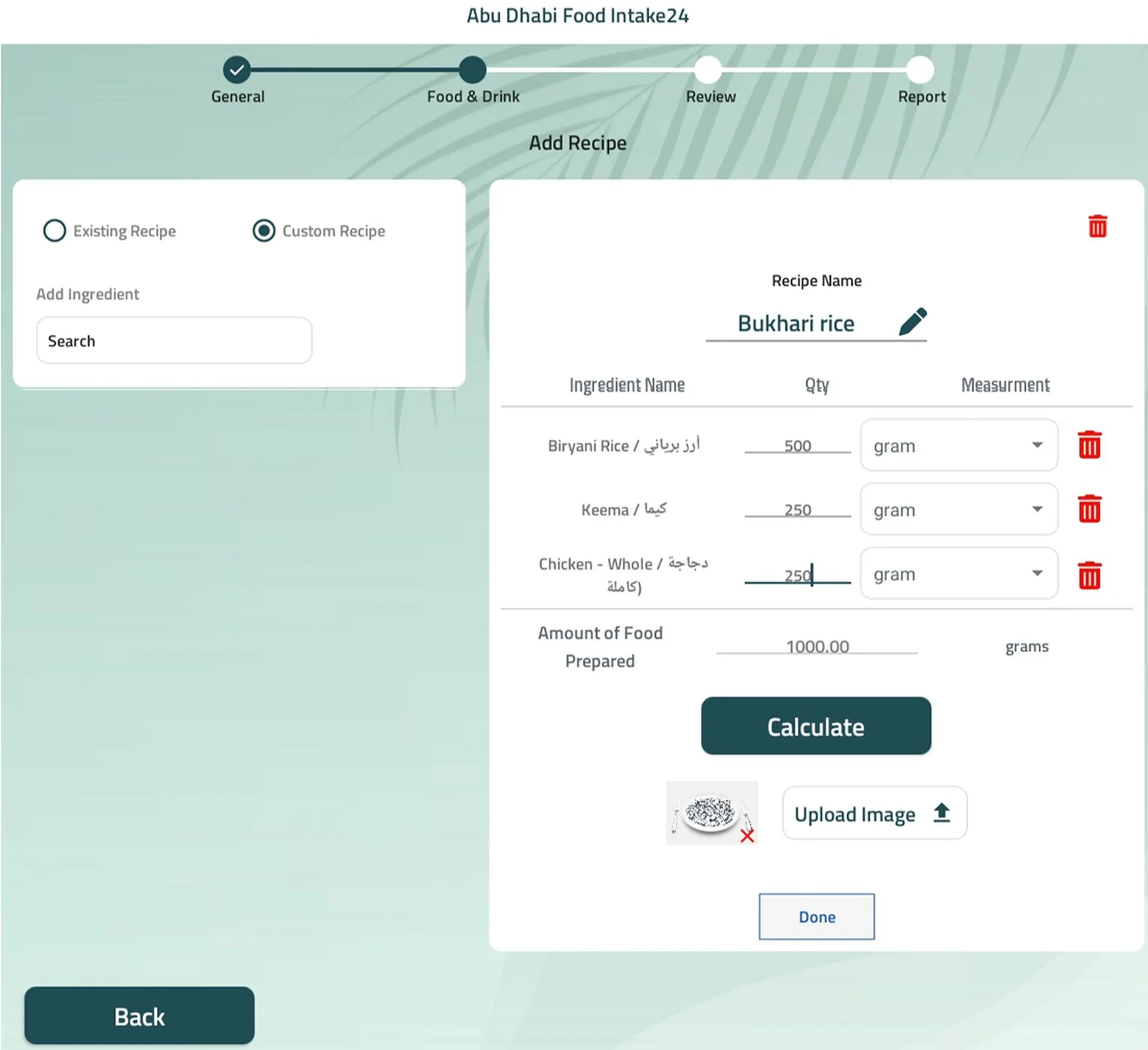
Custom recipe entry interface in Abu Dhabi Food Intake24. The application enables users to create a custom recipe by searching for and adding individual ingredients from the food composition database, specifying the quantity and measurement unit for each ingredient, and entering the total amount of food prepared. An optional image of the prepared dish can also be uploaded. The nutrient composition of the recipe is automatically calculated and added to the 24 h dietary recall. Figure has been reproduced from Abu Dhabi Food Intake 24, licensed under the Apache License, Version 2.0 (the “License”); you may not use this file except in compliance with the License.

If a participant’s food item is not available in the database, the application allows users to add it using the “Add Recipe” feature (Figure 3). Upon selection, users can choose between existing and custom recipes.

Under the existing recipe option, users can search the database for preloaded recipes, review their ingredients, remove irrelevant components, and add new ingredients to create a modified version. The preloaded recipes were collected as part of a previous nutrition survey of a representative sample of Emirati families (44). These recipes were linked to the food composition database to enable nutrient calculation. Under the custom recipe option, users can create a new recipe by selecting individual ingredients directly from the database. In both cases, users specify the quantity of each ingredient separately before submitting the recipe. Once saved, the newly created recipe becomes searchable within the application. To preserve data integrity and prevent alteration of verified database entries, user-created recipes are accessible only to the individual user who created them and are not shared across other users. As in the case of the Abu Dhabi Food Consumption Survey, which is a household-based, interviewer-led survey, the created recipe is accessible when searching for the food intake of the same household members.

**Figure 4:**
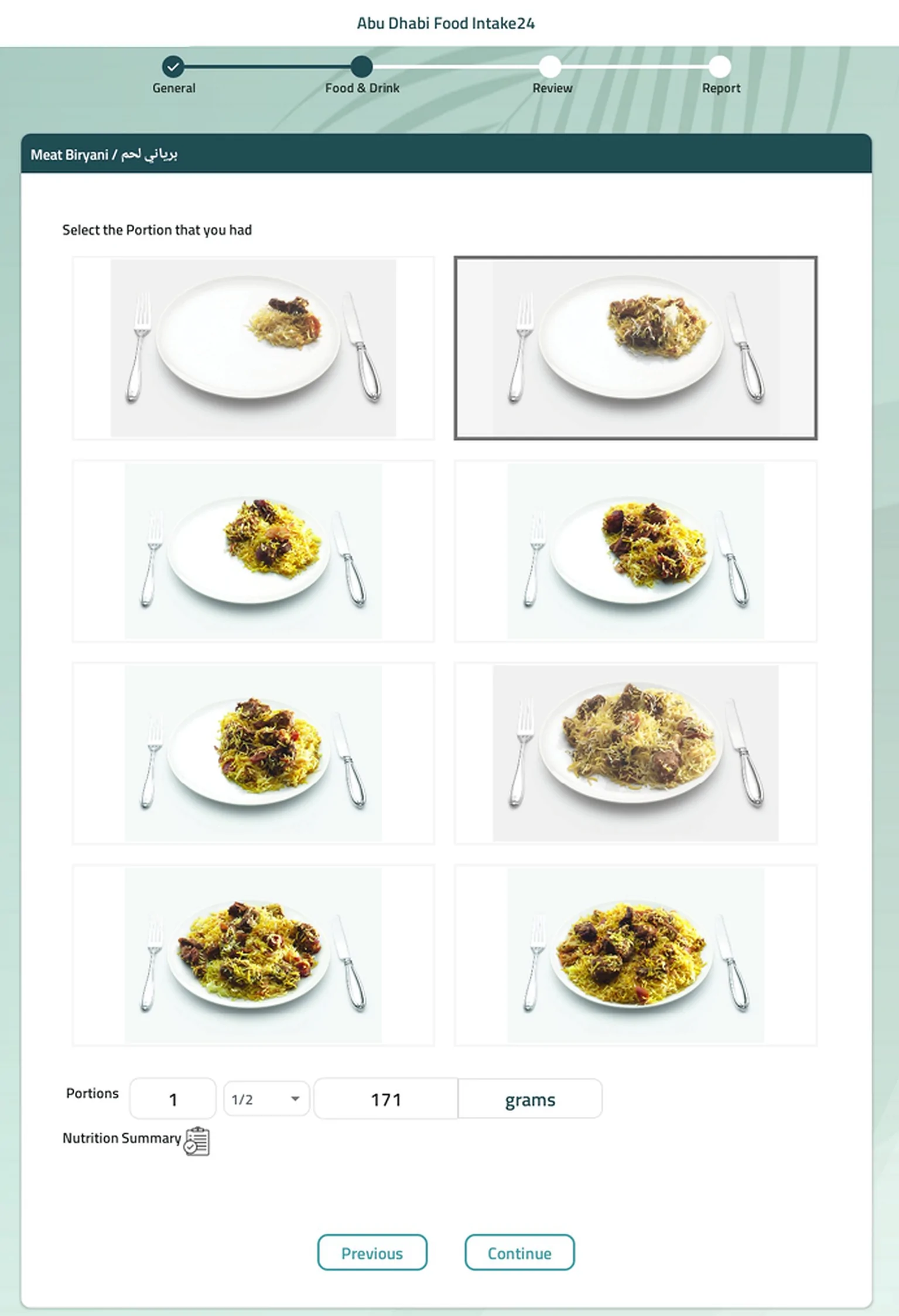
Portion size estimation interface in Abu Dhabi Food Intake24. The application uses a UAE food photograph atlas to assist users in estimating portion sizes. For each selected food item, a series of standardized portion-size photographs is displayed, allowing users to choose the image that best represents the amount consumed. The corresponding portion weight (g) is automatically assigned and linked to the food composition database for nutrient intake calculation. Figure has been reproduced from Abu Dhabi Food Intake 24, licensed under the Apache License, Version 2.0 (the “License”); you may not use this file except in compliance with the License.

Estimation of food portion size is facilitated using the UAE Photographic Atlas of Food Portions (45), which has been integrated into the Abu Dhabi Food Intake24. The food atlas includes photographs of 115 food items commonly consumed in the UAE, the Arabian Gulf, and the Middle East, presented either as a series of 8 images of increasing portion sizes or as a range of sizes similar to those typically available in the market. Figure 4 depicts sample food portion sizes available in the Abu Dhabi Food Intake24.

#### 2.1.3 Nutritional intake calculations and output

After completing the 24-h dietary recall and submitting all reported food items, users are prompted to enter their physical activity level. Based on the entered anthropometric and demographic information, the application automatically calculates Body Mass Index (BMI) and Estimated Energy Requirements (EER). These values are incorporated into the individualized nutrition report.

The final step of the application generates a comprehensive nutrition report, which calculates the macro- and micronutrient content of the completed dietary log and compares the results with Dietary Reference Intakes (DRIs) to facilitate interpretation. The application provides multiple graphical displays to summarize nutrient intake and includes an export function that allows users to save or share their report. Upon completion, the finalized report is submitted to a secure central server where all study data are stored.

#### 2.1.4 Web portal

The web-based portal serves multiple administrative and data management functions. It enables centralized user management, including adding and removing authorized users. The portal also functions as the primary data management platform, allowing review of records submitted through the tablet application to verify completeness and accuracy. This review process serves as an integral component of data quality control. In addition, the portal includes a data export function that allows records to be downloaded in .csv format, facilitating subsequent statistical analysis using standard analytical software. The system generates two primary export files: person-level data and food-level data. Person-level data include participants’ demographic characteristics, responses to the integrated questionnaires, and total energy and nutrient intakes reported separately for each 24-h recall (e.g., Recall 1 and Recall 2), conducted on non-consecutive days. Food-level data contain detailed information on all reported food items, including food type, quantities, preparation method (when applicable), eating occasion (e.g., meal or snack), concurrent activity with eating (e.g., watching television, using a mobile phone), eating location, and whether the food was prepared at home or outside the home.

### 2.2 Pilot survey

The Abu Dhabi Food Consumption Survey was designed to recruit a representative sample of 3,920 households across the three regions of the Emirate of Abu Dhabi. The survey was approved by the United Arab Emirates University Human Research Ethics Committee on 20 January 2024 (Protocol No. ERH2023_2669_05) and the Department of Health-Abu Dhabi (Protocol #: DOH/CVDC/2023/1192). As the first phase of the survey, a pilot study was conducted to evaluate the field implementation of the Abu Dhabi Food Intake24 system before its use in the main survey. The pilot study was conducted in April 2024 by five trained interviewers in Abu Dhabi and Al Ain. The third region of the Abu Dhabi Emirate (Al Dhafra) was not included because of logistical constraints.

Participants in the pilot study were recruited from households in the study areas using a convenience sampling approach. Eligible participants were individuals aged 7 years and older, consistent with the target population of the Abu Dhabi Food Consumption Survey. At least two eligible participants were recruited from each household. Interviewer-administered 24-h dietary recalls were collected electronically using Lenovo Tab P11 tablets. The Abu Dhabi Food Intake24 application automatically records the start and end times of each participant’s visit. The recorded duration represents the entire visit, including questionnaire administration, anthropometric measurements, and the 24-h dietary recall, rather than the dietary recall interview alone.

Before data collection, all interviewers completed standardized training on the multiple-pass 24-h dietary recall method and its implementation using the Abu Dhabi Food Intake24 application. The training covered the five-step multiple-pass recall process, probing techniques to improve the completeness of dietary reporting, portion-size estimation, and standardized procedures for recording foods, beverages, and recipes. The training program included an introductory session on the survey objectives and the Abu Dhabi Food Intake24 application delivered by the principal investigators, followed by a technical session conducted by the software development company, a hands-on practice session facilitated by the project research coordinator, and practical training in standardized anthropometric measurement procedures, including the measurement of height and weight. Interviewers subsequently completed three to five practice interviews with family members or other volunteers to demonstrate competency before commencing fieldwork. An interviewer guide was developed to support the standardized use of the application and the multiple-pass 24-h dietary recall protocol throughout the study.

Following completion of the training, five interviewers conducted the pilot study using the Abu Dhabi Food Intake24 application. Feedback from the pilot study informed iterative refinements to the application before its implementation in the Abu Dhabi Food Consumption Survey, during which all interviewers used the same updated version of the application. Throughout data collection, ongoing support was provided by an assigned member of the research team and through a dedicated WhatsApp group that included both the research team and the software developers to facilitate the timely resolution of technical and operational issues.

### 2.3 System usability evaluation

#### 2.3.1 Participants

Following implementation of modifications based on pilot study feedback, interviewers involved in the Abu Dhabi Food Consumption Survey, including those who participated in the pilot study, were invited to complete a system usability evaluation of Abu Dhabi Food Intake24 as part of its iterative development. A total of 35 interviewers (aged 22–50 years) participated in the evaluation. All interviewers were female except one. They held at least a bachelor’s degree in nutrition or a related field, while some were senior undergraduate or graduate students in nutrition and other health sciences disciplines. As interviewers were recruited in phases, the duration of their field experience and the number of interviews completed before the usability evaluation varied. The system usability evaluation was approved by the United Arab Emirates University Social Sciences Research Ethics Committee on 27 May 2025 (Approval No. ERSC_2025_6944). Interviewers completed the System Usability Scale (SUS) questionnaire (46) after gaining practical experience using the Abu Dhabi Food Intake24 application during fieldwork following the pilot study and throughout the main survey. The sample of 35 interviewers exceeded the number of evaluators typically reported in digital health software usability studies (2–19 evaluators) (47).

#### 2.3.2 Instrument

System usability was assessed using the System Usability Scale (SUS), a widely used tool for evaluating the usability of products and systems (46). The SUS consists of 10 statements with answers rated on a 5-point Likert scale (1 = strongly disagree to 5 = strongly agree). It includes both positively worded items (e.g., “I thought the system was easy to use”) and negatively worded items (e.g., “I found the product unnecessarily complex.” The scoring follows standard SUS procedures: for odd-numbered items (1, 3, 5, 7, and 9), 1 is subtracted from the user’s answer, and 5 is subtracted from the responses to the even-numbered items (2, 4, 6, 8, and 10). The adjusted scores are summed and multiplied by 2.5 to yield a total score ranging from 0 to 100. The sum of all item contributions was then multiplied by 2.5 to yield the final SUS score, ranging from 0 to 100 (48). A higher final SUS score indicates better usability (49). The questionnaire was administered in English using Google Forms and completed anonymously by the participants.

#### 2.3.3 Statistical analysis

Statistical analyses were performed using IBM SPSS Statistics version 31.0 (IBM Corp., Armonk, NY, United States). The Shapiro–Wilk test was used to assess the normality of the System Usability Scale (SUS) score distribution. Data from the pilot study were presented as frequencies, means, and standard deviations. Descriptive statistics were calculated to summarize SUS scores used for the system usability evaluation, including frequencies, mean, standard deviation (SD), median, interquartile range (IQR), minimum and maximum values, and skewness and kurtosis. These measures were used to describe the central tendency, variability, and distributional characteristics of the usability scores.

## 3 Results

### 3.1 Pilot study

A total of 39 households comprising 98 participants were included, of whom 75 (76.5%) were adults and 23 (23.5%) were children and adolescents. Participants were recruited from Abu Dhabi (42.9%) and Al Ain (57.1%) between April and May 2024. The demographic characteristics of the pilot study participants are presented in Table 1. Among adult participants, 49.3% had completed university education.

**Table 1:** Characteristics of the participants of the pilot study (*n* = 92).

Characteristic	Adults (≥19 years) (*n* = 75)	Children and adolescents (8–18 years) (*n* = 23)	Total
Participants, *n* (%)	75 (76.5)	23 (23.5)	98 (100.0)
Age, mean (SD), years	37.8 (15.3)	13.4 (3.2)	—
Gender, *n* (%)			
Female	57 (76.0)	14 (60.9)	71 (72.4)
Male	18 (24.0)	9 (39.1)	27 (27.6)
Education, *n* (%)			
Illiterate	3 (4.0)	—	3 (4.0)
Completed primary school	6 (8.0)	—	6 (8.0)
Completed secondary school	29 (38.7)	—	29 (38.7)
Completed university	37 (49.3)	—	37 (49.3)
Region	Households, *n* (%)	Participants, *n* (%)	
Abu Dhabi	19 (48.7)	42 (42.9)	
Al Ain	20 (51.3)	56 (57.1)	
Total	39 (100.0)	98 (100.0)	

The pilot study evaluated the functionality of the Abu Dhabi Food Intake24 system. Interviewers identified several issues related to data saving during household interviews, reviewing and verifying recorded entries, and searching the server-based food composition database. Feedback collected during the pilot was used to iteratively refine the application (Table 2). The resulting improvements included enhancements to data saving, keyword-based food search, and record preview features. The pilot study findings informed iterative refinements to the application before and during implementation in the Abu Dhabi Food Consumption Survey.

**Table 2:** Pilot study findings, impact on data collection, and refinements implemented in the Abu Dhabi Food Intake24 system.

Issue identified during pilot testing	Impact on data collection	Refinement implemented
Difficulties saving data during household data collection.	Risk of incomplete or lost interview records during fieldwork.	Saving functionality was improved to enhance data reliability during household interviews.
Limited ability to review and verify recorded entries for data errors.	Reduced ability to detect and correct data entry errors before submission.	Preview and review functions were enhanced to facilitate verification of entered data.
Difficulties searching the server-based food composition database, including locating preloaded food lists and adding new recipes.	Reduced efficiency and increased interview time when identifying foods and recipes.	Keyword-based search and food search functions were refined to improve efficiency during interviewer-administered data collection.
Additional interviewer feedback on workflow and application performance.	Identified opportunities to improve system usability and workflow.	Iterative refinements were implemented throughout the pilot and the Abu Dhabi Food Consumption Survey, resulting in the first stable operational version of the Abu Dhabi Food Intake24 system.

### 3.2 System usability evaluation

All 35 responded and completed the usability questionnaire. Table 3 shows the System Usability Scale (SUS) scores for the Abu Dhabi Food Intake24 software characteristics based on responses from 35 interviewers. The Shapiro–Wilk test indicated that SUS scores were normally distributed (*p* = 0.337). The mean SUS score was 69.9 (95% CI: 66.0–73.9), with a standard deviation of 11.6 and a standard error of 2.0, reflecting moderate variability in usability perceptions. Moreover, SUS scores ranged from 45.0 to 97.5.

**Table 3:** System Usability Scale (SUS) score probability distribution for the Abu Dhabi Food Intake24 software (*n* = 35).

Characteristic	Abu Dhabi Food Intake24 software
SUS score Shapiro–Wilk, *p*-value	0.337
SUS score, mean (95% C.I.)	69.9 (66.0–73.9)
SUS score, standard deviation	11.6
SUS score, standard error	2.0
SUS score, median	67.5
SUS score, interquartile range	62.5–80.0
Minimum	45.0
Maximum	97.5
Skewness	0.104
Kurtosis	0.396

The median score was 67.5, and the interquartile range was 62.5–80.0. Skewness (0.104) and kurtosis (0.396) values were close to zero, further confirming the normal distribution of the SUS scores.

A lower mean score was found for items related to system inconsistencies (e.g., “Do you find any inconsistencies in the system?”) and those required prior learning before use (e.g., “I need to learn a lot of things before I can get going with this system.”). On the other hand, higher mean scores were reported for items reflecting user confidence (e.g., “I feel confident using the system”) and perceived ease of learning (e.g., “I would imagine that most people would learn to use this system very quickly”).

### 3.3 System refinement based on user evaluation feedback

Feedback from users after the pilot study and the system usability evaluation was incorporated to enhance the functionality of Abu Dhabi Food Intake 24. Several modifications were implemented, including user interface enhancements to facilitate smoother navigation, the ability to edit entries at multiple stages within the application, and the addition of a final preview and summary page before submission.

Improvements were also made to search engine functionality. In the original version, when the interviewer evaluated the Abu Dhabi Food Intake24 application, users were required to enter the exact spelling and word order of food items, as stored in the databases, which posed challenges, particularly for multi-word food items (e.g., “hard-boiled egg”). The updated search engine allows users to enter partial keywords (e.g., “boiled” or “egg”), and the system retrieves all relevant items containing those terms, regardless of word position. This enhancement reduced the time required to identify food items during data collection and improved overall interview efficiency.

Additional features were added to enhance the user experience. A nutrition profile preview function was added for each selected food item, allowing users to view the nutritional profile before making a selection. The system also generates an individualized nutritional intake summary report based on the completed 24-h recall and compares it against the Dietary Reference Intakes. This feature provides personalized feedback to respondents regarding their dietary intake.

## 4 Discussion

This study describes the development, pilot testing, and usability evaluation of Abu Dhabi Food Intake24, a culturally adapted digital 24-h dietary recall application developed to support large-scale dietary assessment in the United Arab Emirates (UAE). The application was designed to assess dietary intake and eating practices using the multiple-pass 24-h dietary recall method and integrates international, regional, and UAE-specific food composition databases to reflect the country’s multicultural dietary patterns. Pilot testing informed iterative refinements to the application, and the subsequent usability evaluation demonstrated that the software achieved an acceptable level of usability among trained interviewers.

The pilot study played an important role in the iterative development of Abu Dhabi Food Intake24. Qualitative feedback from interviewers identified several practical issues related to data entry, record review, food search functionality, and data export. These findings informed successive refinements to the application, including improvements to the food search engine, editable data-entry functions, preview features, and redesign of the data export format, supporting its implementation under real-world conditions in the Abu Dhabi Food Consumption Survey.

Following these refinements, the software achieved a mean System Usability Scale (SUS) score of 69.9 (SD = 11.6), which falls within the acceptable usability range according to established SUS benchmarks and is slightly above the benchmark mean of 68 reported for digital health applications (47). The interquartile range (62.5–80.0) indicates that most interviewers rated the application within the acceptable usability range, although the wider range of individual scores (45.0–97.5) reflects variability in user experience. This variability may be explained by differences in interviewers’ field experience because interviewers were recruited for the Abu Dhabi Food Consumption Survey at different stages and therefore had varying durations of application use before completing the usability evaluation. Differences in digital literacy may also have influenced usability ratings, although these data were not formally collected. The system usability evaluation of the Abu Dhabi Food Intake24 findings demonstrated an acceptable level of usability for interviewer-administered dietary assessment while highlighting opportunities for continued refinement of the user interface and user experience.

Item-level responses to the SUS indicated that interviewers generally found the application easy to learn and felt confident using it after training. Lower ratings for system consistency and certain application functions identified areas for improvement, particularly the food search engine and navigation features. These findings were consistent with the feedback obtained during the pilot study and informed subsequent refinements implemented before and during the Abu Dhabi Food Consumption Survey. The qualitative pilot feedback and quantitative usability evaluation further supports the value of combining iterative field testing with standardized usability assessment during software development.

Abu Dhabi Intake24 shares several conceptual features with established web-based 24-h recall systems such as the Automated Self-Administered 24-h Dietary Assessment Tool (ASA24) (13), and the United Kingdom Intake24 (50). Abu Dhabi Intake24 utilizes a structured multiple-pass recall approach, automated nutrient calculation, and centralized data management to facilitate large-scale dietary data collection. However, unlike ASA24, which is primarily based on United States food composition databases and dietary patterns, Abu Dhabi Intake24 integrates international (USDA), regional (Kuwaiti), and UAE-specific food composition data to better reflect the foods commonly consumed by the UAE’s multicultural population. The application incorporates the UAE Photographic Atlas of Food Portions to support portion-size estimation for locally consumed foods (45). These adaptations are particularly important because dietary assessment tools developed for Western populations may not adequately capture traditional dishes, mixed recipes, or culturally specific food preparation methods commonly consumed in the Gulf region. In addition, iterative refinements based on field interviewer feedback, such as enhanced keyword-based search functionality and preview features, were implemented to improve efficiency during interviewer-administered data collection.

Usability scores reported for Abu Dhabi Food Intake24 are comparable to those reported for other digital dietary assessment systems. United Kingdom Intake24 (7) reported a SUS score of approximately 83, while myfood24 (12) achieved a score of approximately 74. However, both are web-based tools. Application-based systems such as NutriDiary (27), the “Eat and Track app” (51), and DitEetIk! (28) reported mean SUS scores ranging from 66 to 74. Although direct comparisons should be interpreted with caution due to differences in study populations, study designs, and evaluation methods, these findings suggest that Abu Dhabi Food Intake24 performs similarly to other contemporary digital dietary assessment tools while addressing the unique cultural and dietary practices of the UAE population.

The present study also highlights the importance of cultural adaptation during the development of digital dietary assessment tools. Large food composition databases increase the likelihood that foods consumed by diverse populations are represented but may also make food searching more challenging. Consequently, continuous refinement of food-search algorithms and user interface design is essential to support efficient data entry. The iterative modifications implemented during this study illustrate how user feedback can improve software functionality and usability before large-scale implementation. Although Abu Dhabi Food Intake24 was developed to support self-administered dietary recalls, it was implemented as an interviewer-administered tool in the present study because its self-administered use has not yet been formally validated.

Abu Dhabi Food Intake24 is among the first culturally tailored digital 24-h dietary recall systems developed for the Gulf region. Although validation against established dietary assessment methods is needed, the application addresses an important gap by incorporating international, regional, and UAE-specific food composition databases that reflect the country’s multicultural dietary patterns. This comprehensive database enhances the tool’s relevance to the UAE population but also increases the complexity of food search (52), highlighting the importance of efficient search algorithms and continued refinement of the user interface. Despite the relatively high costs of development and maintenance, digital dietary assessment tools can be cst-effective for large-scale, population-based nutrition surveys.

### 4.1 Strengths

This study has several strengths. Abu Dhabi Food Intake24 is the first culturally adapted digital multiple-pass 24-h dietary recall application developed specifically for dietary assessment in the UAE. The integration of international, regional, and local food composition databases provides comprehensive coverage of foods and beverages consumed by the country’s multicultural population. The Abu Dhabi Food Intake24 system estimates dietary intake for 31 dietary components, including energy, macronutrients, dietary fiber, cholesterol, fatty acids, water, caffeine, vitamins, and minerals. This nutrient profile is suitable for assessing dietary intake and adherence to dietary recommendations in nutritional epidemiology and clinical nutrition research.

The incorporation of the USDA multiple-pass dietary recall methodology, a validated approach for collecting 24-h dietary recall data (53), provides a standardized framework for dietary data collection. In addition, iterative refinements based on pilot testing and usability evaluation enhanced the application’s functionality throughout its implementation in the Abu Dhabi Food Consumption Survey. Furthermore, the application was deployed within a large-scale population-based food consumption survey, providing valuable real-world experience that informed its ongoing development.

### 4.2 Limitations

First, interviewers were recruited at different stages of the survey, resulting in varying levels of experience using Abu Dhabi Food Intake24, which may have influenced the usability scores. Second, although usability testing was conducted following the pilot study, it was not repeated after the final version of the application was completed, limiting the assessment of usability after all iterative refinements had been implemented. Third, we did not collect formal information on interviewers’ digital literacy or previous experience with dietary assessment beyond their educational qualifications and standardized nutrition training. Fourth, as with all 24-h dietary recall methods, Abu Dhabi Food Intake24 is subject to limitations associated with self-reported dietary intake, including memory decay, omission of food items, and potential under- or over-reporting of food and food portion sizes (54).

A limitation of Abu Dhabi Food Intake24 is that it does not currently estimate nutrient intake from dietary supplements. The inclusion of dietary supplements should be planned for a future version of the platform. Finally, the application has not yet undergone formal validation in the target population. Future research should include validation studies comparing Abu Dhabi Food Intake24 with established dietary assessment methods to evaluate its accuracy in both interviewer-administered and self-administered settings.

## 5 Conclusion

Abu Dhabi Food Intake24 (ADINTAKE24) is a culturally adapted, multiple-pass digital 24-h dietary recall application developed to support dietary assessment in the United Arab Emirates. Pilot testing and system usability evaluation informed the iterative refinement of the application under real-world survey conditions, resulting in acceptable usability among trained interviewers. The application has the potential to support large-scale dietary assessment and food consumption surveys in the UAE. Future research should include a second usability evaluation to assess the impact of the iterative refinements on system usability and formal validation studies comparing Abu Dhabi Food Intake24 with established dietary assessment methods. Expanding compatibility beyond Android tablets, including support for iOS devices, may further improve accessibility and facilitate broader implementation.

## Data Availability

The original contributions presented in the study are included in the article/supplementary material, further inquiries can be directed to the corresponding author.
